# Symptoms of Anæsthesia and Administration of Anæsthetics

**Published:** 1861-02

**Authors:** 


SYMPTOMS OF ANÆSTHESIA AND ADMINISTRATION OF ANÆSTHETICS.—*Lecture IV., Tuesday, May* 22, 1860.—Dr. Richardson, after a brief *résumé* of his last lecture, opened the present lecture by proceeding to explain the phenomena of anæsthesia, and the different actions of anæsthetics. With regard to the class of narcotics that produce their effects by catalysis, of which class chloroform at present stands first on the list, these are absorbed by the blood, and remain for some time in the system; they are gradual in their effects during their action, not only during the successive periods or stages of insensibility, but during those stages also which mark recovery. Those narcotics, again, which produce their effects by negation, and of which we may take carbonic acid gas as an example, are different in their effects, there being no distinctly progressive advancement through defined stages. Insensibility is quickly and suddenly produced by these, and the return to consciousness, when it occurs, is almost immediate and perfect. Dr. Snow describes chloroform as giving four successive degrees in producing its effect upon the patient. The first degree is that of excitement; this stage lasts from one to two or three minutes; it is attended by a resistance of the muscles. The second degree is attended by tendency to, or by actual convulsion and violent action of the heart; the pulse is always quick and small, and the patient often gets violent and very loquacious; patients, nevertheless, on their return to consciousness, rarely have any recollection of what they have said or done in this degree. The third degree is attended by the complete annihilation of consciousness and movement; the muscles are relaxed; the pulse is at first full and quick, then moderately slow, until it subsides into the natural pulse; the eyeball is rolled up; "and it is worthy of notice that when this last fact occurs in the administration of any anæsthetic, it is a sure sign that the patient is ready for the performance of an operation." The recovery from this stage generally takes place within four minutes. The fourth degree is attended with very great prostration; the breathing becomes stertorous and noisy; there is an entire paralysis of the body, and, occasionally, spontaneous evacuations. This stage is very dangerous, and is seldom employed unless great relaxation of the muscles is required. Dr. Richardson on this occasion subjected a dog to the vapor of chloroform, and kept the animal in it until the fourth degree had commenced; he then removed the dog, and its stertorous breathing was plainly audible.

Ether has a prolonged first degree, and a very short second; and we can go no further than the third without destroying life; the pulse is excited throughout every stage; the eyeball rolls up in the third degree as in chloroform, but the breathing is more difficult, and on the whole there is a greater appearance of drunkenness than under chloroform.

The administration of amylene is attended with scarcely any excitement, either mentally or physically, the patient sinking rapidly into the third degree. The great difference between chloroform and amylene is, that there is less perfect abolition of consciousness during the administration of the latter. (Vide Lecture I.)

Carbonic oxide, fumes of Lycoperdon, and Dutch liquid, produce nearly the same phenomena as chloroform. Olefiant gas is more slow; and opium differs in having a long first degree, no second degree, but a very long third degree.

The reason of anæsthetics producing sickness seems to be that they are brought by the blood to the mucous membrane of the stomach, there to be eliminated and evacuated; their presence there causes the irritation. Dr. Richardson related an experiment he had made with reference to this: he killed a rabbit with chloroform, and afterwards produced complete anæsthesia on a mouse by merely confining it in a chamber with the rabbit's stomach, thereby thoroughly proving the presence of chloroform. Sulphuric ether, carbonic oxide, fumes of Lycoperdon, Dutch liquid, and monochlorurетted Chloride of Ethyle, have the same effects as chloroform in producing sickness. Amylene differs, there frequently being no vomiting, and when it does occur, it is very slight. It is almost an impossibility to procure an anæsthetic that will not cause sickness; but if we could get one, it would be invaluable, as sometimes the vomiting is very serious. In one case on record, a patient vomited for no less a time than sixteen hours. With regard to a remedy for this sequence of anæsthesia, opium is the best.

After taking all these anæsthetics and their phenomena into our consideration, we must come to the conclusion that there are none at present equal to chloroform; not because it is the least dangerous, but because it is easily inhaled, and because there is never that perfect rest and quiet with other anæsthetics that there is with chloroform. With ether, the patient constantly begins to rally before the operation is finished. Dr. Snow was once asked why he used chloroform in preference to sulphuric ether, or other anæsthetics less dangerous? His answer was, "that he used it for the same reason that he used a lucifer match instead of the old tinder box; not because it was the least dangerous, but because it was the most applicable."

A question that is very frequently asked is—Will eventually any other anæsthetic supersede chloroform? Dr. Richardson is of opinion that that may be the case; Dutch liquid and amylene—the latter would be the best if we could get it pure—are, in fact, nearly equal to chloroform. There is no doubt but that great advantages would be derived by entirely excluding both chlorine and oxygen from the composition of an anæsthetic.

In reference to the question, whether it is necessary to examine a patient previous to administering chloroform? Dr. Richardson expressed an opinion that it was very necessary; differing in that respect from Dr. Snow, who said that no examination was at all necessary, and for his (Dr. Snow's) part, he always left it to the Surgeon indiscriminately; his practice teaching him that when an operation was justifiable, narcotism was justifiable. Out of 4,000 cases in which he had administered chloroform, only one death occurred. For all this we must remember that an examination should be made, as it can do no harm, and may be the means of preventing evil. Further, Dr. Richardson did not approve of the administration of chloroform in simple operations—as, for instance, the extraction of a tooth—as a general rule, but only in great operations, where operative proceeding is necessary for the saving of life.

Previous to the administration of an anæsthetic, attention should be paid to the diet of the patient; it should be a rule, never to administer the vapor until two hours after food has been taken into the stomach, or severe vomiting may occur. The last meal that is taken previous to inhalation should be a light one: a glass of sherry or brandy may be administered without any harm, previous to the inhalation of the chloroform; in fact, it will do good if the patient is nervous. The proper position for the patient is a semi-recumbent position; and after the patient is once placed in the position he is to occupy, he should not be moved, as movement is apt to cause faintness. In one case that Dr. Richardson witnessed of an operation on the lip under the influence of chloroform, they laid the patient down, causing him to faint immediately. The reason of this is, that change of position always alters the pulse. Thus, the pulse which would give in an upright position seventy-five beats in a minute, would give in a sitting position seventy, and from sixty to sixty-four, in the horizontal position.

The mode of administration of chloroform was important. Professor Simpson never administered it but on a handkerchief or napkin: but this is a very dangerous practice, and can not be too strongly reprehended; it is objectionable on many grounds, for some people take in more air than others in inspiration, and it is impossible to tell how much the patient is inhaling. By this method, also, a large portion of the chloroform escapes, and mingles with the atmosphere, to the annoyance of the operator; but, perhaps, the greatest argument against this method of inhalation is, that out of the whole number of deaths that have taken place during or from the administration of chloroform, there are only ten fatal cases where a proper inhaler was used; in all the other cases the chloroform was administered on a towel, handkerchief, or napkin. In one case of a boy that died recently while under the influence of chloroform, it appears that the operator laid a handkerchief containing chloroform over the boy's face while proceeding to operate. The boy died from deficiency of air and excess of chloroform, both causes acting together.

Having come to the conclusion that chloroform ought to be administered by an apparatus, the question arises, which apparatus is the best? Dr. Sibson's mouthpiece is a very good one, but Dr. Snow's inhaler is the very best. It consists of an inner and outer case; the outer case is filled with water, and by this simple arrangement the chloroform is always kept at a uniform temperature, which is a great point, but the chief feature of this inhaler is, that it regulates the inhalation; it is of little consequence what amount of chloroform is put into the instrument, as the patient can breath no more than 5 per cent. at any time. Two drachms is a very good quantity to put in to commence with, and is generally sufficient to produce anæsthesia; afterwards, eight or ten minims added every three or four minutes, will be quite sufficient to keep the patient in the narcotized state.

In one case in which Dr. Richardson administered chloroform, he kept the patient (a tall man) in an anæsthetic state for thirty-seven minutes, and only used three drachms of chloroform from beginning to end; had he wished to keep the patient insensible for that time by the handkerchief process, he would probably have had to use at least two ounces.

Ether is best administered on a sponge. Dr. Hayward was the first to introduce this method.

Amylene is usually administered in quantities of four drachms; frequently, however, from eight to ten drachms will be required to produce the desired effect. Dr. Snow's chloroform inhaler is the best that can be used for this anæsthetic.

Dr. Richardson here particularly requested the attention of his auditors to the following necessary rules to be observed by the operator in the administering of an anæsthetic.

In chloroformization, the first and second degrees are the most dangerous; in the first degree, be careful to watch the effects of the chloroform in relation to production of cough and irritation of the throat; if these occur, remove the chloroform for a moment. In the second stage, observe for convulsion; and if that is great, remove the chloroform. As the third stage comes on, observe the eye of the patient, for the first indications of the ball rolling up. The finger must be kept all through on the pulse; and if any cessation is felt, withdraw for a moment the narcotic. In giving chloroform, every sense must be trained; the eye, to see expression and action; the touch, to determine the pulse; and the ear, to seize as acutely as possible the breathing of the patient, and to ascertain whether the respiration is in any way impeded.

Lastly, there are two indications which show that the patient is ready for operation; when the eyeballs are turned upwards, and there is no flinching on touching the eye, insensibility is complete, and the operator may proceed, *cito et tuto.—Dr. Richardson's Lectures, reported for the Dental Review.*

